# Layout-aware text extraction from full-text PDF of scientific articles

**DOI:** 10.1186/1751-0473-7-7

**Published:** 2012-05-28

**Authors:** Cartic Ramakrishnan, Abhishek Patnia, Eduard Hovy, Gully APC Burns

**Affiliations:** 1 Information Sciences Institute, University of Southern California, 4676 Admiralty Way, Suite 1001, Marina del Rey, CA, 90292-6695, USA; 2 Computer Science Department, University of Southern California, 941 Bloom Walker, Los Angeles, CA, 90089-0781, USA

## Abstract

**Background:**

The Portable Document Format (PDF) is the most commonly used file format for online scientific publications. The absence of effective means to extract text from these PDF files in a layout-aware manner presents a significant challenge for developers of biomedical text mining or biocuration informatics systems that use published literature as an information source. In this paper we introduce the ‘Layout-Aware PDF Text Extraction’ (LA-PDFText) system to facilitate accurate extraction of text from PDF files of research articles for use in text mining applications.

**Results:**

Our paper describes the construction and performance of an open source system that extracts text blocks from PDF-formatted full-text research articles and classifies them into logical units based on rules that characterize specific sections. The LA-PDFText system focuses only on the textual content of the research articles and is meant as a baseline for further experiments into more advanced extraction methods that handle multi-modal content, such as images and graphs. The system works in a three-stage process: (1) **Detecting contiguous text blocks** using spatial layout processing to locate and identify blocks of contiguous text, (2) **Classifying text blocks into rhetorical categories** using a rule-based method and (3) **Stitching classified text blocks together in the correct order** resulting in the extraction of text from section-wise grouped blocks. We show that our system can identify text blocks and classify them into rhetorical categories with Precision^1^ = 0.96% Recall = 0.89% and F1 = 0.91%. We also present an evaluation of the accuracy of the block detection algorithm used in step 2. Additionally, we have compared the accuracy of the text extracted by LA-PDFText to the text from the Open Access subset of PubMed Central. We then compared this accuracy with that of the text extracted by the PDF2Text system, ^2^commonly used to extract text from PDF. Finally, we discuss preliminary error analysis for our system and identify further areas of improvement.

**Conclusions:**

LA-PDFText is an open-source tool for accurately extracting text from full-text scientific articles. The release of the system is available at http://code.google.com/p/lapdftext/.

## Background and motivation

The field of Biomedical Natural Language Processing (BioNLP) is maturing, with specific fields of software development in response to user requirements: *e.g.*, links between databases and literature, better tool interactivity and integration and the development of high-quality NLP resources [1,2]. NLP techniques such as Named Entity Recognition [3] and Semantic Relation Extraction [4] have been shown to be very useful to biologists studying protein-protein interactions [5] and Gene-Disease-Phenotype relations [6]. Given the ubiquity of the ‘Portable Document Format’ (PDF) as a means of distributing scientific publications and since access to information in full-text documents is vital for developing effective text-mining applications [7], it is essential to the general BioNLP community that developers of such applications can extract the textual content from PDF files accurately with open-source tools. Many past biomedical text mining studies have used either the abstracts of scientific papers [8-11] or relatively small collections of full-text articles sampled from the Open Access subset of PubMed Central [12]. It is likely that certain content of journals of interest in a particular task is not distributed as a part of the Open Access subset.

A long-standing promise of BioNLP has been to help accelerate the vital process of literature-based biocuration, where published information is carefully translated into the knowledge architecture of biomedical databases, using specific BioNLP tools [1,8,13]. The identification of all papers relevant to the specific database being populated can be considered as a document classification problem [12]. Subsequent steps have been cast as Information Extraction (IE) problems that leverage context dependent features [14,15]. A key consideration is that the well-crafted manual workflows, developed by expert curators in biomedical databases, typically use rules based on context and rhetorical structure-dependent clues found only in the full-text of an article. Thus, it is important for the developers of BioNLP applications to have access to an accurate representation of the full-text of papers derived from PDF files, see [16].

Our goal is to provide an open-source software mechanism for automated decomposition and conversion of PDF files of research articles into a simple text format that other NLP groups can easily incorporate into their toolsets. In the most widely used text extraction programs (*e.g.*, Adobe Acrobat, Grahl PDF Annotator, IntraPDF, PDFTron and PDF2Text), the flow of the main narrative from a file may be broken in mid sentence by errors derived from the reading order of individual text blocks and interruptions such as the inclusion of figure captions, footnotes and headers.

The variation in styles and formats of research articles (even within a single journal) can cause errors in terms of the ordering and splicing of text between pages and blocks. Any software that performs such decomposition and extraction should be adaptable with minimal human effort to new styles and formats. Driven by these needs, our system focuses on providing an open source PDF-to-text conversion capability meeting the following requirements: (1) the extraction mechanism should be able to adapt to single-column, two-column or mixed single and double column layouts, (2) extracted text should be error-free and grouped according to specific section headings used in the paper and (3) formatting artifacts such as, headers, footers, figures, tables and floating boxes (used in author summaries) should not interrupt the narrative-flow within each section. Thus, we have developed a three-step approach for extracting text from PDF files. The first step is the identification of contiguous text blocks. The second step is the classification of these text blocks into rhetorical categories (such as ‘Introduction’, ‘Results’ and ‘Discussion’) using logical rules that are easy to generate as ‘decision tables’ in a spreadsheet. The third step utilizes the classification results to ‘stitch’ appropriate text blocks together for extracting the text, while ignoring blocks that contain formatting embellishments so as to minimize flow-disruption of the extracted text. Our system provides programmatic, open-source access to each one (or to all three) of these capabilities for individual files or large collections of files.

## Implementation

### Step 1 - Detecting contiguous text blocks

The first step in LA-PDFText is to identify contiguous text blocks. In addition to the frequently-used two-column and single-column formats, journals also often use a mixed format where the title, authors, affiliation and abstract span the entire page width (single-column format) while all other sections of the article use a two-column format. We have observed these changes in format by manually inspecting papers from all available issues of the journal **Brain Research**. We denote these periodic changes in formatting over the lifetime of a given journal as ‘epochs’.

**Figure 1:**
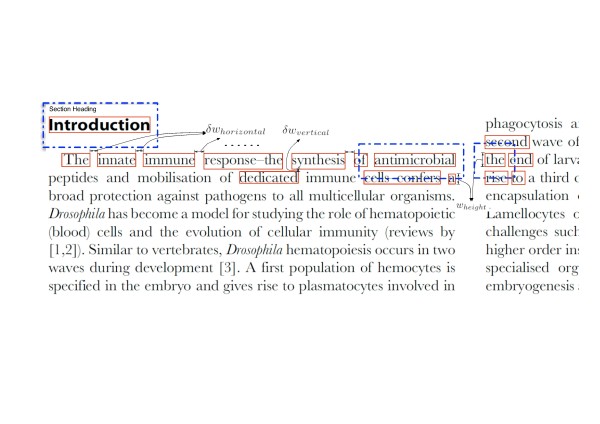
**Block detection per-page parameter computation algorithm.** The image shows the process by which the north–south and east–west parameters for neighboring block subsumption are computed. For an explanation of the symbols shown in the figure please see Table 1.

Our approach to detecting contiguous text blocks starts with detecting ‘word-blocks’ (bounding boxes of words). We use the GPL version of JPedal, an open-source Java PDF library to obtain the bounding boxes of each word in the PDF article (http://www.jpedal.org/). Using this as a starting point, LA-PDFText aggregates word-blocks systematically to build ‘chunk-blocks’ of text while respecting formatting constraints such as two-column *vs.* one-column formatting. As shown in Figure 1, the algorithm for identifying text blocks, functions by coalescing word blocks together that are close enough (based on the spatial statistics of the words’ layout on the page) and share font characteristics. The algorithm computes proximity automatically on a per-page basis giving it flexibility in dealing with varying formats both within a single page and across pages.

**Table 1:** Per page word block parameters symbols and their definitions

**Parameter Symbols**	**Definitions**
**w**_**height**_	Word block height
**δw**_**horizontal**_	Horizontal space between words
**δw**_**vertical**_	Vertical space between words
**max**^**i**^_**f**_**(δw**_**height**_**)**	Most Popular Word block height in a page *i*
**max**^**i**^_**f**_**(δw**_**horizontal**_**)**	Most Popular Horizontal space between word blocks in a page *i*
**max**^**i**^_**f**_**(δw**_**vertical**_**)**	Most Popular Vertical space between word blocks in a page *i*
**ϕ**_**EW**_ **= max**^**i**^_**f**_**(δw**_**height**_**) + max**^**i**^_**f**_**(δw**_**horizontal**_**)**	east–west word block expansion parameters in page
**ϕ**_**NS**_ **= max**^**i**^_**f**_**(δw**_**height**_**) + max**^**i**^_**f**_**(δw**_**vertical**_**)**	north–south word block expansion parameters in page

Figure 1 is an example of how the block detection algorithm decides which word blocks to coalesce. Examples of the parameters δw_horizontal_, δw_vertical_ and w_height_ are shown in Figure 1. The distributions of these parameter values are calculated for each page and the most popular values for these parameters are chosen from these distributions to calculate ϕ_EW_ and ϕ_NS_. We intentionally do not use most popular word width since biomedical text uses many long words and the most popular word width will make ϕ_EW_ too large thereby making the block subsumption algorithm too greedy. Consider the words ‘Introduction’ in the section heading, the word ‘antimicrobial’ in the first line of the first column and the word ‘the’ in the third line of the second column in Figure 1. Each word-block (shown in red) is surrounded by an expanded bounding-box (shown using a blue dotted line). All word-blocks (shown in red) that intersect with this expanded bounding-box are treated as words blocks to be merged. The block merge procedure is a greedy algorithm and will combine a section heading, subheading and the sections content into a single block based on the ϕ_EW_ and ϕ_NS_ parameters.

**Figure 2:**
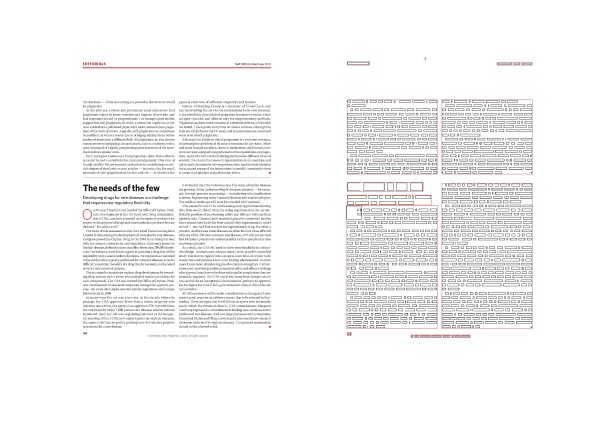
**Flexibility of the block identification algorithm.** The image shown on left of the figure is taken from page 2, with two distinct articles, of the **Nature** editorial Volume 466 Issue no. 7303. The image on the right is an example of the debug output generated by LA-PDFText. Our block detection algorithm identifies the text blocks in the right column of the article page as distinct blocks allowing the subsequent block classification step of the system to apply rules that treat these blocks as parts of different articles.

To examine the flexibility of our block detection algorithm, we use a PDF file of the **Nature** editorial in Volume 466 Issue no. 7303 (Figure 2). This issue contains 3 editorials, and the second page contains part of the second editorial along with the third, separated by a horizontal line. LA-PDFText was able to accurately identify, classify and extract text from the editorial PDF file.

### Step 2 - Classifying text blocks into rhetorical categories

The next phase of LA-PDFText is based on ‘DROOLS’, a business rule management system and an enhanced Rules Engine implementation, ReteOO, based on the Rete algorithm [17] tailored for the Java language distributed as part of the open-source JBoss Enterprise Platform (http://labs.jboss.com/portal/jbossrules/). DROOLS provides a way for the LA-PDFText user to declaratively specify characteristics of a text block that make it a part of a particular section in the paper. We include the rule files for two epochs within the PLoS Biology dataset in both the DROOLS format as well as Microsoft Excel (Additional files 1,2 and 3).

### Step 3 - Stitching classified text blocks together in the correct order

The final goal of LA-PDFText is to accurately extract the text of any given section(s) in the correct sequence. As an implementation of this capability the last component of the LA-PDFText iterates over the classified blocks and stitches the classified blocks together to produce contiguous sections along with section and sub-section headings appropriately demarcated. LA-PDFText provides mechanisms to output the text of these PDF as XML formatted using PubMed Central’s OpenAccess DTD.

## Results

We have evaluated the three steps of our system independently of each other. In the following sections we will present our evaluation methods for each of the three steps of LA-PDFText and their results.

### Step 1 - Detecting contiguous text blocks - evaluation

In order to evaluate the effectiveness of spatial segmentation of each PDF page into text blocks, we manually segment each page in our experimental dataset to produce the ideal segmentation of each paper. We then count the number of edit operations (deleting and adding blocks) required to transform the manually segmented papers into to the segmentation predicted by our software. The ideal segmentation of a paper is one that does not require any deletion, addition or splitting of segments in order to retrieve the text from the segments in the correct order. We use the following guidelines in the manual segmentation process: (1) segments should be created in such a way as to facilitate sequence-preserving text extraction, (2) segments should be rectangular and (3) section headings and sub-headings should be marked as distinct segments from the body of their corresponding sections. Our algorithm creates images of each page of the input PDF showing the word block boundaries and the segment boundaries (Figure 2). To explain the evaluation process further, we present the following sample situations (Table 2) that describe block configurations produced by LA-PDFText. In each case, we describe edit operations applied to the manually segmented page and their corresponding cost. The results of this evaluation are presented in Additional file 4: Tables S4, S5, S6 and S7 under the column titled ‘Spatial Segmentation Score’. In the ideal case a paper segmented into blocks by LA-PDFText should have a spatial segmentation score of zero, indicating that it is perfectly segmented with respect to the manual segmentation.

**Table 2:** Example scenarios describing conversion operations and their corresponding costs

**System Output**	**Operation in Gold Standard Representation**	**Cost**
Block is split	Split the gold standard block into the required number	1
Big block is subsuming n small blocks	Delete the involved blocks in the gold standard and add one big block	n + 1
n block are intersecting	Delete all the blocks in the gold standard, whose area is common with the intersecting blocks, in the system output	Number of blocks deleted from gold standard +1

### Step 2 - Classifying text blocks into rhetorical categories - evaluation

The rule based segment classifier component of our software is instrumented to produce color-coded segments depending upon the type of section to which each segment belongs. This color-coding is used in the manual evaluation to count the number of segments of each section that were correctly classified (true positives; TP), those that were incorrectly classified (false positives; FP) and those that were missed by the rule engine (false negatives; FN). Thus, we can calculate the Precision (P), Recall (R) and F1 metrics to evaluate the classification accuracy using the following metrics:

(1) $$P=\frac{TP}{TP+FP}\;R=\frac{TP}{TP+FN}\;F1=\frac{2*P*R}{P+R}$$

The results of this manual evaluation are reported in Additional file 4: Tables S4, S5, S6 and S7 under the column titled ‘Block Classification Performance’. Classification Precision, Recall and F1 scores are averaged across all volumes and presented in Table 3 in a per-section basis.

**Table 3:** Per-section Precision (P), Recall(R), and F1 scores for section classification

**N**	**Section Parts**	**P**	**R**	**F1**
Paper Title		1.000	0.966	0.983
Authors		0.987	0.906	0.945
Abstract	Heading	1.000	1.000	1.000
	Body	0.988	0.883	0.933
Introduction	Heading	1.000	0.988	0.994
	Body	0.876	0.915	0.895
Results	Heading	1.000	1.000	1.000
	Body	0.948	0.912	0.930
	Sub-heading	0.947	0.843	0.892
Methods	Heading	1.000	1.000	1.000
	Body	0.992	0.927	0.958
	Sub-heading	1.000	0.982	0.991
Discussion	Heading	0.987	1.000	0.993
	Body	0.946	0.924	0.935
	Sub-heading	0.917	0.885	0.901
Figure Legend		0.986	0.840	0.907
References	Heading	1.000	0.988	0.994
	Body	0.532	0.632	0.578
Supporting Information	Heading	0.988	1.000	0.994
	Body	0.946	0.224	0.362
** Macro Average**		**0.956**	**0.888**	**0.910**

### Step 3 - Stitching classified text blocks together in the correct order - Evaluation

PDF2Text is a widely used approach to extract text from PDF files. However, it is unable to distinguish between formatting embellishments and the main narrative of a scientific article. PDF2Text treats the entire document as one string, introducing errors within individual sentences, at column breaks and page breaks. LA-PDFText classifies each text block and (provided the classification is accurate) stitches text blocks belonging to the same section together, in order to extract contiguous rhetorical sections of the input articles. We have compared the text extraction capabilities of both systems to evaluation step 3 of LA-PDFText. Although PDF2Text is a simpler tool to use, we evaluate LA-PDFText’s text extraction capability against that of PDF2Text to show the benefit of our three-stage approach to text extraction.

**Figure 3:**
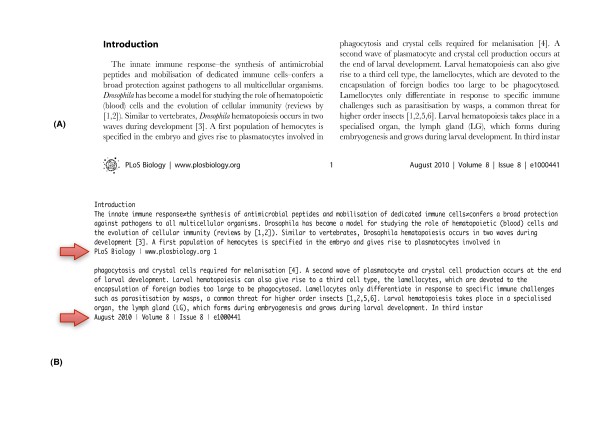
**Text Flow Interruptions.** The image (A) in the figure above is a snippet of text extracted from the corresponding PDF file (shown in image B) by PDF2Text. The red arrows on the extracted text mark a break in text flow generated by PDF2Text owing to its inability to discount formatting embellishments like footers. Our evaluation of text extraction accuracy quantifies the effect of such flow-interruption on the quality of the output text produced by both PDF2Text and LA-PDFText.

Figure 3 shows an example of the text extraction produced by PDF2Text where the string “PLoS Biology ∣ http://www.plosbiology.org 1” interrupts the preceding sentence. The interruption is precisely the sort of error that is unacceptable in many applications of BioNLP, especially those contributing to biocuration. Our evaluation therefore seeks to quantitatively capture the notion of ‘flow-disruption’. Our strategy is based on comparing text extracted by PDF2Text and LA-PDFText for a given set of research articles, against the text extracted from the XML representation of that paper within the Open Access Subset. We chose PLoS Biology articles at random from volumes 5, 6, 7, and 8 for this evaluation. These XML files contain the full-text of their corresponding articles, along with the necessary markup that demarcates each section of the paper. The XML does not contain headers and footers present in the original PDF.

**Figure 4:**
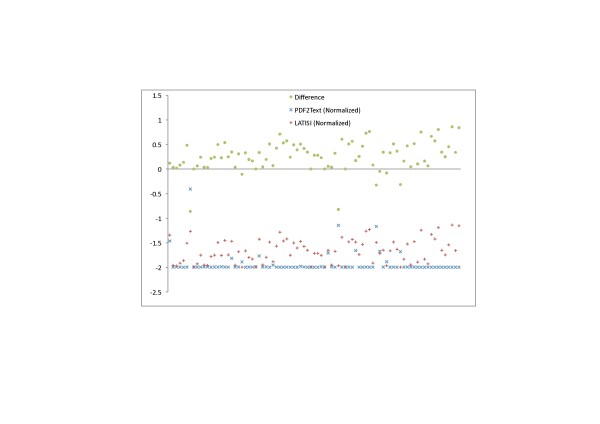
**Text Flow Evaluations.** The graph above shows the relative alignment cost of LA-PDFText and PDF2Text with respect to the gold standard. Each green dot represents the difference between the normalized alignment scores of LA-PDFText and PDF2Text for one paper in the PLoS Biology dataset. + markers show normalized alignment scores produced by LA-PDFText and - markers show normalized alignment scores produced by PDF2text. Results indicated that LA-PDFText extracts text with better alignment scores with respect to the gold standard than PDF2Text for 91% of the documents tested (p < 0.001).

We use a variant of the Needleman-Wunsch algorithm [18] to compute alignment costs for text extracted by both algorithms against text obtained from the Open Access XML for each paper. The Needleman-Wunsch algorithm uses dynamic programming to perform a global alignment on two sequences and using linear gap penalties. Our variant of this algorithm treats the Open Access text as a sequence of sentences and computes the cost of aligning sentences generated by LA-PDFText and PDF2Text with sentences in the Open Access text. The algorithm uses a gap penalty of −10, a mismatch penalty of −1 and a match reward of 5. Computed alignment costs for each paper are normalized by dividing them by the number of sentences in the Open Access version of the text for that paper. The resulting number can be interpreted as the ‘average per-sentence alignment cost’ for a given paper. The difference between normalized costs produced by both methods is plotted in the graph shown in Figure 4. A number greater than zero indicates that LA-PDFText produced a higher alignment score with respect to the Open Access text than PDF2Text for a particular paper. A number less than zero indicates that PDF2Text produced a better alignment score. Figure 4 shows that only 7 out of 86 documents extracted by LA-PDFText (shown using +) produce a poorer alignment score with the Open Access text than PDF2Text (shown using -). In other words, in 91% of the cases LA-PDFText outperforms PDF2Text (p < 0.001). It should be noted that the text extracted by LA-PDFText used in this experiment still contain errors introduced due to sections that have not been classified into any rhetorical categories (recall errors). Despite these classification errors LA-PDFText extracts text with fewer flow interruptions resulting in higher accuracy of extracted text than PDF2Text.

## Discussion

LA-PDFText is designed to be a baseline system as a precursor for further improvements to the block detection, classification and text extraction stages. In this section, we discuss the results of each stage of LA-PDFText presenting error analyses and identify proposed future improvements.

### Step 1 - Detecting contiguous text blocks

LA-PDFText’s block detection algorithm is fairly accurate (see Spatial Segmentation Score in Additional file 4: Tables S4, S5, S6 and S7). Over the PLoS Biology dataset, block detection results in alignment scores with mean (μ) = 9.5 and standard deviation (σ) = 5.7. The algorithm depends on the accuracy of JPedal at identifying word blocks. Although it is expected that using a commercial version of JPedal will reduce these scores and improve block detection, we want LA-PDFText to be available for use without the need for users to purchase the commercial version (although we may release a version of our systems that can also work with the commercial version of JPedal).

### Step 2 - Classifying text blocks into rhetorical categories

**Figure 5:**
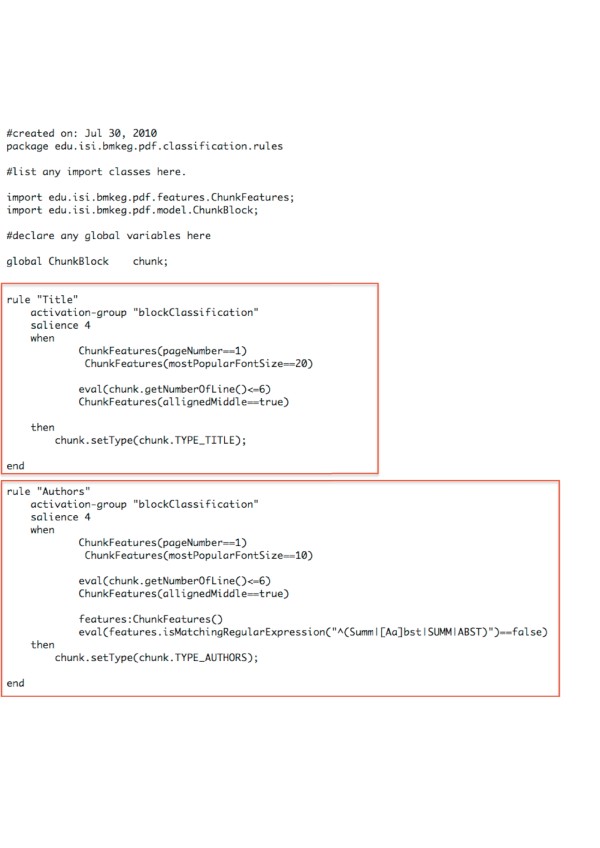
**Sample Rule File Listing.** The figure shows examples of DROOLS Rules for block classification. DROOLS files meant for two epochs within the PLoS Biology dataset are available as a part of the software distribution accompanying this paper. They can also be downloaded from http://code.google.com/p/lapdftext/. The two files included are named epoch_7Jun_8.drl and epoch_5_7May.drl and are located in a folder called `rules' in the base directory of the installation. Experiments reported in this paper have been conducted using these rules for the block classification stage. These files are also included as supplementary material for this paper.

We have designed the segment classification component of LA-PDFText using a rule-based approach so as to make the system more flexible and easily adaptable for use with various journal formats. The classification results (Table 3) are based on rule files (see Figure 5) that we designed in roughly a single working day. The goal of our project is to provide a PDF-extraction library that can be customized for specific uses by BioNLP developers. Thus, we have provided a mechanism that requires a relatively small time investment from developers to classify PDF-based text blocks with suitable levels of accuracy. The software distribution includes a Microsoft Excel based ‘decision table’ which can be used to fill in values for features of blocks that cause rules to ‘fire’ and generate an appropriate labels for blocks. The ‘decision table’ mechanism will also allow non-programmers to specify rules for block classification.

We have identified specific errors in the rules that were responsible for poor performing categories (Table 3). Within PLoS Biology, the classification recall for the section titled ‘Supporting Information’ is only 0.224 (Table 3). Close inspection of our dataset reveals that most supporting information sections contain figure legends, which belong to two categories namely ‘Figure Legends’ and ‘Supporting Information’. The system correctly classifies the blocks as figure legends but not as supporting information. Both the precision and recall of the section titled ‘References’ are 0.532 and 0.632 respectively (Table 3). We attribute the low score to the fact that the font used in tables in many papers is the same as that used in references. Since our baseline rule-set did not contain a rule to identify tables they get wrongly identified as references resulting in poor recall and precision.

### Step 3 - Stitching classified text blocks together in the correct order

The quality of text extraction is best determined by the usability of the text by downstream text mining applications. We have presented evaluations that show the ability of LA-PDFText to extract text with fewer flow interruptions than text extracted by PDF2Text. It should be noted that the evaluation of text extraction was done on full text of papers explicitly to contrast LA-PDFText with PDF2Text. LA-PDFText also provides the user with the additional capability to extract text on a per-section basis; a capability that PDF2Text does not support.

## Related work

Since the introduction of Portable Document Format in 1993 and the widespread development of online journals in the late 1990s, many archival documents published earlier have been scanned and converted into PDF. Furthermore, the scientific community and publishers have adopted PDF as the *de facto* standard format for scientific communication. In this paper we therefore do not focus on the Optical Character Recognition (OCR) problem but instead assume that we are given PDF documents that include the text, fonts, images, and 2D vector graphics. We are primarily concerned with related work in development of PDF extraction systems that support BioNLP work in the academic community.

Discovering the logical structure of documents is a well-studied problem. However most past efforts were aimed a logical-structure discovery [19,20] and not explicitly aimed at text extraction from PDF documents. Furthermore, these past efforts used OCR to produce images of document pages, which are then segmented and the segments are classified to discover logical structure. Summers et al. present a survey of methods for the document-logical-structure discovery problem [21]. While some methods surveyed by the author perform joint segmentation and classification, other methods separate these steps into distinct phases. Certain methods use a multi-level form of bounding boxes as the basis of their joint segmentation and decision-tree based classification [19] for logical-structure discovery. All of the above methods are aimed at inducing some hierarchical representation of the document content from document images. The method presented in this paper uses bounding boxes as well but separates the segmentation and classification phases.

One recent effort aimed at recovering the logical structure of the scholarly articles using Nuance OmniPage 16 to identify bounding boxes of words [22]. The bounding box information is represented in XML that includes markup indicating each line and paragraph within the input PDF. The words, lines and paragraph information along with font information of each word are used as features to train a Conditional Random Field (CRF) [23] model to classify each line into one of 23 predetermined classes corresponding to rhetorical categories. The method proposed in [22] relies on a commercial tool; a feature we seek to avoid here. The authors performed tests on two datasets: one comprising 40 scientific papers in the field of computer science and the other from their previous work comprising 211 **Association of Computing Machinery** (**ACM**) papers. We downloaded the second dataset^3^ and manually inspected the PDF documents. We observed that formatting across the 211 papers from **ACM** is fairly regular using a two-column format. In contrast, we have tested LA-PDFText on articles from the journal **Brain Research** spanning volumes 1 to 1155. Manually we have identified 10 significant formatting changes from 1966 to 2007. In order to deal with all articles within PubMed,^4^a PDF extraction system will have to deal with these formatting variations. The system developed by [22] also produces XML similar to the LA-PDFText system and can therefore produce text on per-section basis. Upon close inspection of their results, we observed that formatting embellishments interrupt the flow of text extracted by their system in much the same way as it is in PDF2Text’s results. We believe that this is due to the fact that their system does not use a rule-based classification of text blocks, and may not be flexible enough to incorporate this change without substantial effort in feature engineering and retraining.

PDF extraction was used in the Mouse Genome Informatics (MGI) system to generate text input for text-mining software *in-situ*[16]. They used a collection of commercial software (IntraPDF, PDFTron and specifically ProMiner) to extract text from PDF files but did not describe the process or outcome in detail, making it difficult to compare with our current work. Another toolset of particular interest is the Utopia documents platform [24,25]. Utopia uses PDF as the base framework for constructing an entire toolset within the familiar architecture of a paper. As a first step, the Utopia system performs the text extraction process with a high accuracy, but it does so directly within the rubric of the Utopia system. Our system is a library that provides low-level control of multiple components of the text extraction process and is designed specifically for use by other text mining developers.

## Conclusion & future work

LA-PDFText is built using non-commercial components, making it freely available under the LGPL license. We believe that it is a very useful tool for the BioNLP community owing to its flexibility and adaptability to a variety of journal formats with minimal rule-development effort. We plan to extend this work by extracting text and structure from tables [26], graphs, figures [27] and citations (C [28]). The systems framework is designed in a modular fashion and can incorporate different methods for block detection and block classification. LA-PDFText will be put to immediate use in the development of a variety of biocuration applications. The next version of LA-PDFText will output annotations in compliance with ontologies such as Annotation Ontology [29,30] and ontologies about bibliographic records, citations, evidence and discourse relationships.

## Software verification

In addition to open-source software distribution of LA-PDFText, we also provide the data set that was used in the evaluation presented in this paper (see Additional file 4). During our evaluation process each phase of our systems three-stage process produces intermediate files meant specifically for use by developers to monitor performance. For instance, the block classification phase produces images each page showing color-coded word blocks grouped using chunk block bounding boxes. This has been an invaluable tool for debugging rule files used in the classification process. Further details about verifying our systems output are forthcoming at the project page listed below. Our code contains unit tests that show how to programmatically invoke our system in all its modes of operation. We invite the reader to download the data set from the location indicated in the supplemental file and reconstruct our evaluation.

## Availability and requirements

Project name: LA-PDFText – Layout-Aware Text Extraction from Full-text PDF of Scientific Articles

Project home page: http://code.google.com/p/lapdftext/

Current Version: 1.7

Operating system: MacOSX 10.6.7, Linux and Windows XP

Programming language: Java 1.6

Other requirements: none.

License: GNU General Public License

## **Endnotes**

^1^ Average taken over all class labels in the section classification task

^2^http://download.cnet.com/BatchConvert-PDF2Text/3000-2248_4-75147475.html

^3^http://wing.comp.nus.edu.sg/downloads/keyphraseCorpus/NUSkeyphraseCorpus.zip

^4^http://www.ncbi.nlm.nih.gov/pubmed/

^5^https://wiki.birncommunity.org/display/NEWBIRNCC/SciKnowMine/

## Competing interests

The authors declare that they have no competing interests.

## Authors’ contributions

GAPCB formulated the idea behind LA-PDFText. CR, AP & GAPCB designed and created LA-PDFText. Authors CR and AP re-engineered and modularized the block detection algorithm. AP implemented the rule-based classification using the latest version of DROOLS. CR conducted the manual evaluation of the block detection and the block classification. CR designed the text accuracy evaluation scheme. Authors GAPCB and EH advised on the evaluation methodology. CR implemented and engineered the text evaluation. CR and GAPCB wrote the paper. All authors read and approved the final manuscript.

## Supplementary Material

Additional file 1 Sample block classification rule file ‘epoch_5_7May.drl’. This file contains the rules for block classification for PLoS Biology articles in issue 5 to the May articles in issue 7 in DROOLS format. This rule-file can be used in conjunction with the LA-PDFText application available at http://code.google.com/p/lapdftext/ click here for file

Additional file 2 Sample block classification rule file ‘epoch_7Jun_8.drl’. This file contains the rules for block classification for PLoS Biology articles in issue 7 from June to those in issue 8 in DROOLS format. This rule-file can be used in conjunction with the LA-PDFText application available at http://code.google.com/p/lapdftext/ click here for file

Additional file 3 Sample block classification rule file ‘epoch_7Jun_8.csv’. This file contains the rules for block classification for PLoS Biology articles in issue 7 from June to those in issue 8 in CSV format. This rule-file can be used in conjunction with the LA-PDFText application available at http://code.google.com/p/lapdftext/ click here for file

Additional file 4 Contains supplemental Table 4, 5, 6 and 7 Click here for file
