# Supplementary material for: Layout-aware text extraction from full-text PDF of scientific articles
Source: Source Code Biol Med. 2012 May 28;7:7. doi: 10.1186/1751-0473-7-7 (PMC3441580; doi:10.1186/1751-0473-7-7)
Supplement: Additional file 4 — Contains supplemental Table 4, 5, 6 and 7 [file 1751-0473-7-7-S4.doc]

# Supplementary Material

The following tables contain Precision Recall and F1 Scores measuring the block classification accuracy of our system for the data set used in our experiments. This data is available at <http://code.google.com/p/lapdftext/downloads/detail?name=EvaluationData2.zip&can=2&q>= and <http://code.google.com/p/lapdftext/downloads/detail?name=EvaluationData1.zip&can=2&q>=.

| Document ID | Block Classification Performance | | | Spatial Segmentation Score |
| --- | --- | --- | --- | --- |
|  | Precision | Recall | F1 |  |
| 0050001 | 1.0000 | 0.7600 | 0.8636 | 9 |
| 0050002 | 0.9968 | 0.9062 | 0.9493 | 41 |
| 0050013 | 0.8333 | 0.7353 | 0.7813 | 3 |
| 0050014 | 0.9245 | 0.7538 | 0.8305 | 11 |
| 0050016 | 0.9500 | 0.9766 | 0.9631 | 17 |
| 0050017 | 0.5625 | 0.5538 | 0.5581 | 1 |
| 0050063 | 0.9828 | 0.8507 | 0.9120 | 5 |
| 0050066 | 0.9348 | 0.8431 | 0.8866 | 4 |
| 0050096 | 0.9452 | 0.9583 | 0.9517 | 7 |
| 0050097 | 0.9512 | 0.9286 | 0.9398 | 9 |
| 0050124 | 0.9091 | 0.8333 | 0.8696 | 4 |
| 0050137 | 0.9268 | 0.7755 | 0.8444 | 10 |
| 0050156 | 0.9892 | 0.9020 | 0.9436 | 11 |
| 0050163 | 1.0000 | 0.8491 | 0.9184 | 2 |
| 0050192 | 0.9620 | 0.9500 | 0.9560 | 8 |
| 0050197 | 0.9487 | 0.8605 | 0.9024 | 1 |
| 0050219 | 0.9574 | 0.7759 | 0.8571 | 7 |
| 0050220 | 0.9841 | 0.8267 | 0.8986 | 3 |
| 0050244 | 0.9677 | 0.8571 | 0.9091 | 2 |
| 0050246 | 0.9821 | 0.8209 | 0.8943 | 5 |
| 0050282 | 0.8608 | 0.8718 | 0.8662 | 5 |
| 0050284 | 0.9828 | 0.9194 | 0.9500 | 9 |
| 0050321 | 0.9403 | 0.8630 | 0.9000 | 5 |
| 0050322 | 0.9692 | 0.8289 | 0.8936 | 4 |

Table 1 PLoS Biology Articles From Volume 5. The precision, recall, and F1 scores reported in this table are manually calculated by counting the number of true positive, false positive and false negative block classifications.

| Document ID | Block Classification Performance | | | Spatial Segmentation Score |
| --- | --- | --- | --- | --- |
|  | Precision | Recall | F1 |  |
| 0060011 | 0.9839 | 0.8971 | 0.9385 | 3 |
| 0060017 | 0.9865 | 0.8488 | 0.9125 | 5 |
| 0060045 | 0.9326 | 0.8830 | 0.9071 | 11 |
| 0060050 | 0.5641 | 0.5238 | 0.5432 | 6 |
| 0060074 | 0.9872 | 0.7700 | 0.8652 | 13 |
| 0060079 | 0.9792 | 0.7833 | 0.8704 | 8 |
| 0060131 | 0.9792 | 0.8785 | 0.9261 | 9 |
| 0060135 | 0.9661 | 0.7500 | 0.8444 | 9 |
| 0060158 | 0.9896 | 0.8962 | 0.9406 | 10 |
| 0060159 | 0.9787 | 0.8000 | 0.8804 | 10 |
| 0060191 | 0.9787 | 0.9293 | 0.9534 | 9 |
| 0060194 | 1.0000 | 0.7374 | 0.8488 | 16 |
| 0060209 | 0.9773 | 0.8866 | 0.9297 | 21 |
| 0060214 | 0.9787 | 0.7302 | 0.8364 | 13 |
| 0060219 | 0.9375 | 0.7759 | 0.8491 | 6 |
| 0060247 | 1.0000 | 0.8448 | 0.9159 | 3 |
| 0060249 | 0.9813 | 0.8824 | 0.9292 | 8 |
| 0060263 | 0.9615 | 0.8197 | 0.8850 | 8 |
| 0060264 | 0.9802 | 0.7734 | 0.8646 | 17 |
| 0060299 | 0.9771 | 0.8421 | 0.9046 | 12 |
| 0060300 | 0.9143 | 0.9697 | 0.9412 | 1 |

Table 2 PLoS Biology Articles From Volume 6. The precision, recall, and F1 scores reported in this table are manually calculated by counting the number of true positive, false positive and false negative block classifications.

| Document ID | Block Classification Performance | | | Spatial Segmentation Score |
| --- | --- | --- | --- | --- |
|  | Precision | Recall | F1 |  |
| 1000008 | 0.9753 | 0.7524 | 0.8495 | 14 |
| 1000009 | 0.9890 | 0.9091 | 0.9474 | 18 |
| 1000027 | 0.9706 | 0.8354 | 0.8980 | 6 |
| 1000029 | 1.0000 | 0.8649 | 0.9275 | 4 |
| 1000045 | 0.9651 | 0.9022 | 0.9326 | 9 |
| 1000047 | 0.9655 | 0.8750 | 0.9180 | 6 |
| 1000067 | 0.5862 | 0.3208 | 0.4146 | 14 |
| 1000101 | 0.9825 | 1.0000 | 0.9912 | 2 |
| 1000121 | 0.9886 | 0.8131 | 0.8923 | 9 |
| 1000123 | 0.9882 | 0.8571 | 0.9180 | 13 |
| 1000141 | 0.9213 | 0.7489 | 0.8262 | 14 |
| 1000148 | 0.6667 | 0.6866 | 0.6765 | 16 |
| 1000167 | 0.9737 | 0.8315 | 0.8970 | 8 |
| 1000168 | 0.9894 | 0.8158 | 0.8942 | 18 |
| 1000181 | 0.9762 | 0.7387 | 0.8410 | 11 |
| 1000187 | 0.9915 | 0.8069 | 0.8897 | 14 |
| 1000208 | 0.9875 | 0.8681 | 0.9240 | 10 |
| 1000212 | 0.4771 | 0.5591 | 0.5149 | 10 |
| 1000235 | 0.9861 | 0.7553 | 0.8554 | 10 |
| 1000236 | 0.5235 | 0.6593 | 0.5836 | 7 |
| 1000252 | 0.9877 | 0.8421 | 0.9091 | 9 |
| 1000253 | 0.8533 | 0.6667 | 0.7485 | 11 |

Table 3 PLoS Biology Articles From Volume 7. The precision, recall, and F1 scores reported in this table are manually calculated by counting the number of true positive, false positive and false negative block classifications.

| Document ID | Block Classification Performance | | | Spatial Segmentation Score |
| --- | --- | --- | --- | --- |
|  | Precision | Recall | F1 |  |
| 1000270 | 0.9863 | 0.7273 | 0.8372 | 14 |
| 1000275 | 0.6300 | 0.7241 | 0.6738 | 9 |
| 1000297 | 0.9740 | 0.8065 | 0.8824 | 13 |
| 1000302 | 0.9868 | 0.7979 | 0.8824 | 11 |
| 1000322 | 0.9718 | 0.6900 | 0.8070 | 9 |
| 1000324 | 0.5942 | 0.7664 | 0.6694 | 7 |
| 1000348 | 0.9884 | 0.8333 | 0.9043 | 12 |
| 1000349 | 0.9936 | 0.8960 | 0.9422 | 20 |
| 1000363 | 0.9907 | 0.7431 | 0.8492 | 4 |
| 1000365 | 0.9867 | 0.7629 | 0.8605 | 11 |
| 1000382 | 0.9878 | 0.8617 | 0.9205 | 9 |
| 1000386 | 0.9911 | 0.6416 | 0.7789 | 10 |
| 1000410 | 0.9778 | 0.9263 | 0.9514 | 6 |
| 1000414 | 0.9926 | 0.7283 | 0.8401 | 14 |
| 1000441 | 0.9891 | 0.9579 | 0.9733 | 7 |
| 1000442 | 0.9506 | 0.6581 | 0.7778 | 13 |
| 1000471 | 0.7436 | 0.6127 | 0.6718 | 16 |
| 1000473 | 0.9891 | 0.9479 | 0.9681 | 7 |

Table 4 PLoS Biology Articles From Volume 8. The precision, recall, and F1 scores reported in this table are manually calculated by counting the number of true positive, false positive and false negative block classifications.
